# Comparison of lists of genes based on functional profiles

**DOI:** 10.1186/1471-2105-12-401

**Published:** 2011-10-16

**Authors:** Miquel Salicrú, Jordi Ocaña, Alex Sánchez-Pla

**Affiliations:** 1 Statistics Department, University of Barcelona, Barcelona, Spain; 2 Statistics and Bioinformatics Unit. Institut de Recerca-HUVH, Barcelona, Spain

## Abstract

**Background:**

How to compare studies on the basis of their biological significance is a problem of central importance in high-throughput genomics. Many methods for performing such comparisons are based on the information in databases of functional annotation, such as those that form the Gene Ontology (GO). Typically, they consist of analyzing gene annotation frequencies in some pre-specified GO classes, in a class-by-class way, followed by p-value adjustment for multiple testing. Enrichment analysis, where a list of genes is compared against a wider universe of genes, is the most common example.

**Results:**

A new global testing procedure and a method incorporating it are presented. Instead of testing separately for each GO class, a single global test for all classes under consideration is performed. The test is based on the distance between the functional profiles, defined as the joint frequencies of annotation in a given set of GO classes. These classes may be chosen at one or more GO levels. The new global test is more powerful and accurate with respect to type I errors than the usual class-by-class approach. When applied to some real datasets, the results suggest that the method may also provide useful information that complements the tests performed using a class-by-class approach if gene counts are sparse in some classes. An R library, *goProfiles*, implements these methods and is available from Bioconductor, http://bioconductor.org/packages/release/bioc/html/goProfiles.html.

**Conclusions:**

The method provides an inferential basis for deciding whether two lists are functionally different. For global comparisons it is preferable to the global chi-square test of homogeneity. Furthermore, it may provide additional information if used in conjunction with class-by-class methods.

## Background

With the advent of genomic technologies it has become possible to perform, in a routine manner, experiments which simultaneously analyze the behavior of thousands of genes or proteins in different conditions. A common feature of these studies is that they generate huge quantities of data, which has led to the term "high-throughput" to describe them. There are different types of high-throughput experiments, but here we will refer specifically to the most well known: microarray experiments [1-3]. A typical differential expression study aims to identify genes that are *differentially expressed *under two or more conditions; for instance, healthy (or untreated or wild-type) cells compared to tumor (or treated or mutant) cells. Such experiments often result in long lists of genes which have been selected using a given criterion (for instance a moderated *t*-test followed by a p-value adjustment) to assign them *statistical significance*.

Obtaining one or more lists of genes is only the first step; they must be interpreted in a way that makes biological sense. One common approach is to relate the genes they contain with one or more functional annotation databases, such as the Gene Ontology (GO), or Kyoto Encyclopedia of Genes and Genomes (KEGG). For simplicity we will speak only of GO classes (or categories, or nodes) but many concepts are also applicable to other annotation systems. There are many methods and models for performing this re-processing [4-6]. Two of the most commonly used are *Gene Enrichment Analysis *[7] (EA) and *Gene Set Enrichment Analysis *[8,9] (GSEA). This paper is mainly concerned with the EA approach.

To some extent, EA methods may be considered one-sample procedures in the sense that they try to elucidate the association between a "sample" gene list (e.g. differentially expressed genes in the presence of a tumor type) taken from a given "universe" (e.g. the whole set of genes in the microarray) and a characteristic such as being annotated in a given GO class. In contrast, microarray data may also be used in a context where the goal is mainly the *comparison *of two (or more) "sample" gene lists ([10-13]), such as differentially expressed genes in a sample of induced apoptotic cells *vs *differentially expressed genes in a sample of senescent cells. These lists may share part of their genes, but possibly not all of them. Again, the comparison is made in terms of their annotations in a functional database. A clear example of this approach is the comparison of whole experiments performed by different laboratories, possibly using different microarray technologies, whose resulting gene lists do not always coincide, see for example [14]. Similar or complementary studies that are available may be compared or even combined; thus, the goal of the analysis may be to prove difference or, conversely, to prove similarity.

The statistical model underlying EA and comparison methods based on GO class counts or hits is usually the hypergeometric-multihypergeometric distribution, together with the assumption that the gene "samples" under consideration are taken from a finite universe, e.g. the entire microarray, [15]. This leads to inferential methods mainly based on Fisher's exact test. Sometimes, the underlying model is the less realistic, but simpler to handle, binomial-multinomial distribution, under the assumption that the "samples" are taken from an infinite population. This is the basis of the chi-square approach, e.g. in [16]. In general, the binomial model provides an adequate approximation to the hypergeometric model for sufficiently large marginal frequencies.

Comparison methods typically focus on only one GO class at a time. If multiple classes are considered, the analysis is performed in a class-by-class fashion followed by a correction for multiplicity. An obvious advantage of this class-by-class approach is that classes associated with difference are readily identified. The main drawback of this approach is a loss of power. Controls for multiplicity based on strict criteria such as the family-wise error rate (FWER) unavoidably impose a loss of power, while more permissive criteria such as the false discovery rate (FDR) may be associated to an incomplete type I error control. In other words, the FDR corresponds to the expected proportion of erroneous null hypothesis rejections (false positives) among the total number of observed positives; a good FDR controlling procedure may maintain FDR below a given level, but not maintain the probability of any false positive below a given (significance) level, see for example [17-19]. An alternative approach is testing for difference *jointly *for all classes under consideration. The basis for such an approach in EA is outlined in [20] and a general approach and method is established in [21]. The obvious advantage of the global approach (only one significance test is performed) is a more strict control of type I and II errors. The main drawback is that association or difference is established with respect to a collection of classes, with no identification of those that have a greater influence. Here we present a family of hypothesis tests, and a method based on them, which perform global comparisons but also provide the possibility of combining them with a class-by-class approach, in order to identify the most significant classes.

If *s *denotes the number of GO classes under consideration, note that the common procedures for 2 × *s *frequency tables, such as the usual homogeneity chi-square test, are not appropriate as the GO classes are not mutually exclusive--in the sense that a single gene may be annotated in more than one class. Previous work, [21], established a probabilistic model for the joint distribution of gene hits in GO classes and provided a method for testing the fit of observed annotation frequencies to a given, fully-specified model, in a similar way to the classic goodness-of-fit chi-square test. Here we present an evolution of this method which, under a quite general setting, accounts for *global testing *of the differences between two gene samples, e.g. in an enrichment or experiment comparison context. The analysis may be performed with the objective of either "demonstrating" differences, or conversely demonstrating (near) equivalence, e.g. as an argument in support of the combination of results from two experiments. In this paper we focus in the first approach, i.e. demonstrating difference. This global analysis may be of interest by itself, or may be followed by class-by-class post-hoc comparisons, to determine which classes are more responsible for determining the associations or differences. Under this setting, the global test may provide useful information when sample sizes for specific single classes are small (while global sample size may be adequate). Its type I error level is closer to the nominal level and its power is in general greater than that of the class-by-class approach. For example, at a deep GO level (such as level 10 in the examples below) the global test may detect difference while class-by-class comparisons may be unable to detect any such difference. This may suggest exploring a less specific GO level or even (as the global test provides evidence of the significance of at least one class) to choose as *significant *the class with the smallest p-value.

## Methods

In this section we introduce our method, some notation and the global test procedure, and give a brief description of the associated R software. We conclude this section with the proof of the validity of the global test.

### Decision criteria and algorithm

**Figure 1:**
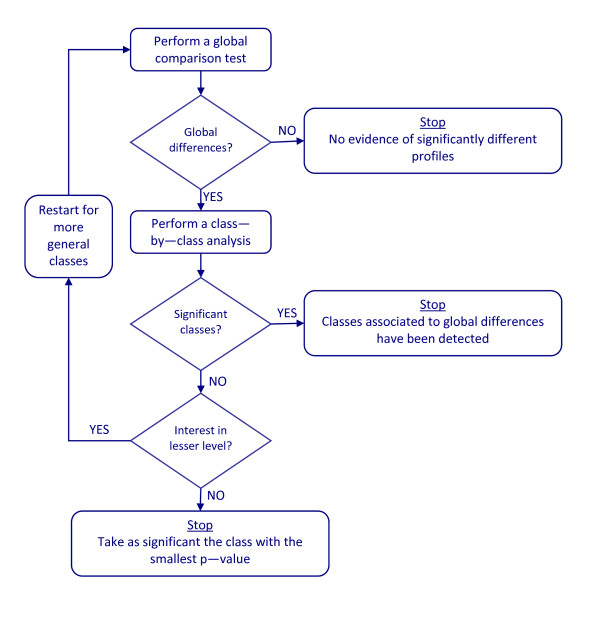
**Flow diagram for the basic algorithm**. Flow diagram to illustrate the method of combining a general profile comparison test and class-by-class analyses.

To complement the information provided by the global test with that from the class-by-class approach, we suggest the method illustrated in Figure 1 and described as follows:

1. At a given GO level, or for a given target set of GO classes (in one or more GO levels), perform a global comparison test. If the global test gives a non-significant result, stop the process: there is no evidence of differences for these GO classes. Otherwise, proceed to the next step.

2. Perform class-by-class testing (e.g. by means of Fisher's exact test^1^, with p-value adjustment) to identify the significant classes. If any of these tests produce significant results, stop the process: significant GO classes have been identified. Otherwise, proceed to the next step.

3. If no significant classes were found in the preceding step (but remember, the global test for differences gave a significant result), either:

(a) Declare as significant the class associated with the lowest unadjusted p-value or, alternatively,

(b) go back to step 2 and test for less specific GO classes, if these classes are still biologically or clinically interesting.

Step 1 is motivated by the need for adequate control on type I errors: by proceeding this way, the type I error of the full procedure is dominated by the type I error of the new global test. Thanks to the safeguard provided in step 1, step 3 may provide an extra possibility to identify truly significant classes. Admittedly, sometimes the class-by-class approach will detect one or more truly significant classes while the global test will give a negative result. But our simulation results indicate that this is a comparatively rare event, and the better power properties of the global test compared to multiple class-by-class testing, particularly in presence of low annotation frequencies, in general largely compensate this small loss of sensitivity.

### Notation and statistical approach

Given a set of GO classes which cut a GO graph at a given (but not necessarily unique) level--or simply a set of "interesting" classes--our approach consists of expanding the original distribution (where one gene can appear in several classes) into a new *expanded *distribution in which each gene belongs to one, and only one, disjoint set. This expanded distribution can be modeled as multinomial or as multihypergeometric, and standard statistical methods can be used to derive the asymptotic distribution of the counts.

We define a *functional profile *as the full vector of counts of the *n *genes in the sample in the *A*_1_, *A*_2_, ..., *A_s _*classes of a given level of an ontology--or, more generally, *s *classes defining a cross section of an ontology, possibly at more than one level. Since single genes may be annotated in more than one class, these counts may sum more than the total number of genes under consideration (if taken as absolute frequencies) or more than one (if taken as relative frequencies). To overcome this problem [21] introduced the concept of an *expanded profile*, defined as the joint frequencies of counts in the set of all possible combined GO classes, which are mutually exclusive. In other words, we distinguish between genes that are annotated *exclusively *in node *A*_1_, genes that are annotated *exclusively and simultaneously *in node *A*_1 _and node *A*_2_, genes that are annotated *exclusively and simultaneously *in nodes *A*_1_, *A*_2 _and *A*_3_, and so on. Expanded profiles should not be confused with plain ("contracted") functional profiles.

**Figure 2:**
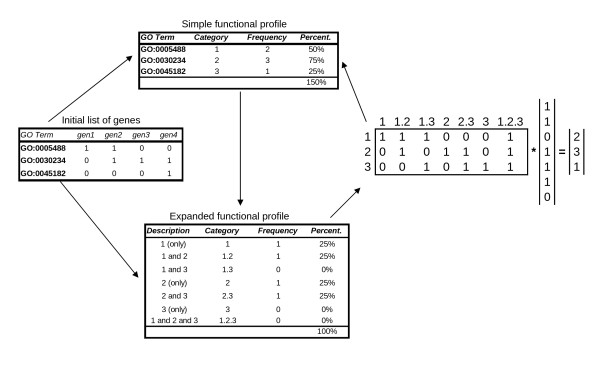
**Basic vs expanded profiles**. A schematic view of basic and expanded functional profiles associated with a list of 4 genes projected at the second level of the MF ontology.

Figure 2 shows the contracted and expanded profiles associated with 4 genes in 3 GO classes.

With these ideas in mind, we establish notation as follows.

The column vector of relative frequencies evaluated over a set of *n *genes is represented by $\hat{P}={\left({\hat{p}}_{1\cdot },{\hat{p}}_{2\cdot },\ldots ,{\hat{p}}_{s\cdot }\right)}^{\prime }$ (or ${\hat{P}}_{n}$ to emphasize that it comes from a "sample" of *n *genes). The "dot" notation ${\hat{p}}_{i\cdot }$ is used to highlight the fact that all the genes annotated in class *i *(but not exclusively in it) have been counted. The term "profile" will indistinctly be used to designate the absolute frequencies, $n{\hat{P}}_{n}$ or the relative frequencies ${\hat{P}}_{n}$ given *n*.

The symbol $\hat{P}$ (or ${\hat{P}}_{n}$) designates an expanded profile, that is, the column vector of relative frequencies

(1) $$\hat{P}={\left({\hat{p}}_{1},{\hat{p}}_{2},\ldots ,{\hat{p}}_{s},{\hat{p}}_{12},{\hat{p}}_{13},\ldots ,{\hat{p}}_{\left(s-1\right)s},{\hat{p}}_{123},\ldots \right)}^{\prime }.$$

Here ${\hat{p}}_{i}$ corresponds to the frequency of genes exclusively annotated in node *A_i_*, ${\hat{p}}_{ij}$ to the frequency of genes exclusively annotated in nodes *A_i _*and *A_j_*, and so on.

All these profiles are taken as sampling realizations of theoretical or population profiles, say *P *and *Q*--or P and Q for expanded profiles.

The dissimilarity between two profiles is measured in terms of their squared Euclidean distance:

(2) $$d\left(\hat{P},\;\hat{Q}\right)=\overset{s}{\underset{i=1}{\sum }}{\left({\hat{p}}_{i\cdot }-{\hat{q}}_{i\cdot }\right)}^{2}.$$

### A new global comparison test

**Figure 3:**
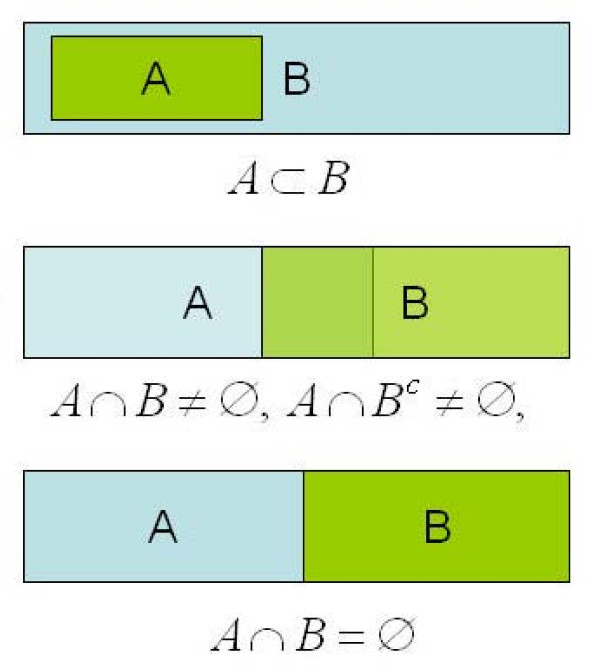
**Relations between lists of genes**. Possible relationships between gene lists to be compared: one list includes the other; two intersecting lists; two non-intersecting lists.

Suppose that we wish to compare the GO profiles of two lists of genes, *A *and *B*, of size *n *and *m*, respectively. Following [22], we note that the lists may share *k *genes, with three possibilities available (see Figure 3):

1. *k *= *n *<*m*, that is *A *⊂ *B*.

2. *k *<*n*, *k *<*m*, that is *A *∩ *B *≠ ∅.

3. *k *= 0, that is *A *∩ *B *= ∅.

Now, let $\hat{P}$ be the sample profile for the first list in a given ontology, and $\hat{Q}$ the corresponding profile for the second list. We have

(3) $$\hat{P}=\frac{k}{n}{\hat{P}}_{0}+\frac{n-k}{n}{\hat{P}}_{1}$$

and

(4) $$\hat{Q}=\frac{k}{m}{\hat{P}}_{0}+\frac{m-k}{m}{\hat{Q}}_{1}$$

where ${\hat{P}}_{0}$ is the profile of the *k *common genes, and ${\hat{P}}_{1}$ and ${\hat{Q}}_{1}$ are the profiles of the non-common genes. Similarly, ${\hat{P}}_{0}$, ${\hat{P}}_{1}$ and ${\hat{Q}}_{1}$ are the corresponding expanded sample profiles.

To test a null hypothesis of profile equality versus an alternative of difference, that is:

(5) $$\begin{array}{c} H_{0}:\;d\left(P,Q\right)=0\\ H_{1}:\;d\left(P,Q\right)>0 \end{array}$$

we can use the fact that, if *H*_0 _is true,

(6) $$V_{n,m}=\frac{nm}{n+m}d\left(\hat{P},\hat{Q}\right)$$

approximately follows the distribution of a linear combination of *s *independent central chi-square-distributed random variables, each one with one degree of freedom:

(7) $$\overset{s}{\underset{i=1}{\sum }}\beta_{i}\chi_{1,i}^{2}.$$

The details of the calculation of the *β_i _*coefficients and in general the proof of the above result are delayed to the end of this section. The result ensures the validity of the following decision criterion: "reject *H*_0 _if *V*_*n*,*m *_>*v *(*α*, *s*)", where *v *(*α*, *s*) stands for the 1 - *α *quantile of the distribution of (7). Likewise, a p-value for (6) can be calculated from (7).

When the population profiles are not equal, the statistic

(8) $${\left(\frac{nm}{n+m}\right)}^{1∕2}\left(d\left(\hat{P},\hat{Q}\right)-d\left(P,Q\right)\right)$$

approximately follows a normal distribution *N *(0, *σ*^2^). As a consequence,

(9) $$d\left(\hat{P},\hat{Q}\right)\pm z_{\alpha }\hat{\sigma }\sqrt{\frac{1}{n}+\frac{1}{m}}$$

defines an approximate 1 - 2*α *confidence interval for *d *(*P*, *Q*), where *z_α _*stands for the 1 - *α *quantile of a standard normal distribution and $\hat{\sigma }$ is a suitable estimate of *σ*. Additionally, expression (8) provides a way to approximately compute the power of the above test. Again, details such as the expression of the variance *σ*^2 ^are considered at the end of this section ("Mathematical details").

### Software

The functionalities described in this paper, together with those in [21], have been implemented in the R package *goProfiles*, available from Bioconductor http://bioconductor.org/packages/release/bioc/html/goProfiles.html.

Package *goProfiles *uses the CRAN package *CompQuadForm *[23] to compute the distribution associated with (7). As an illustration of its use, the R commands associated with the example in the next section are available at http://estbioinfo.stat.ub.es/pubs/goProfiles1_BIF/goProfiles1.htm.

### Mathematical details

From considerations similar to those in [21] we conclude that the asymptotic distribution of $\left(\hat{P},\hat{Q}\right)$ is approximately multivariate normal. More exactly, if

(10) $$D_{n,m}={\left(\frac{n\;m}{n+m}\right)}^{1∕2}\left(\hat{P}-P,\hat{Q}-Q\right)$$

then

(11) $$D_{n,m}\overset{d}{\underset{n,m\to \infty }{\to }}Y\sim N\left(0,\Sigma_{PQ}\right)$$

where the covariance matrix Σ*_PQ _*may be estimated by:

$$\Sigma_{\hat{P}\hat{Q}}=\left(\begin{array}{c} \frac{m}{n+m}A\;\frac{k}{n+m}B\\ \frac{k}{n+m}B\;\frac{n}{n+m}C \end{array}\right)$$

with:

$$\begin{array}{c} A=\frac{k}{n}\Sigma_{{\hat{P}}_{0}}+\frac{n-k}{n}\Sigma_{{\hat{P}}_{1}}\\ B=\Sigma_{{\hat{P}}_{0}}\\ C=\frac{k}{m}\Sigma_{{\hat{P}}_{0}}+\frac{m-k}{m}\Sigma_{{\hat{Q}}_{1}} \end{array}$$

and $\Sigma_{{\hat{P}}_{0}}$, $\Sigma_{{\hat{P}}_{1}}$ and $\Sigma_{{\hat{Q}}_{1}}$ correspond to the covariance matrices associated with the respective profiles ${\hat{P}}_{0}$, ${\hat{P}}_{1}$ and ${\hat{Q}}_{1}$, that have the general form [21]: *σ_ii _*= *p*_*i*· _(1 - *p*_*i*·_) for *i *= 1, ..., *s *and *σ_ij _*= *p*_*ij*· _- *p*_*i*·_*p*_*j*·_, when *i *≠ *j*, for *i*, *j *= 1, ..., *s*.

From the algebraic relation:

(12) $$\begin{array}{llll} d\left(\hat{P},\hat{Q}\right) & ={\left(\hat{P}-\hat{Q}\right)}^{t}\left(\hat{P}-\hat{Q}\right)\; & & \;\\ & ={\left(\hat{P},\hat{Q}\right)}^{t}{ℑ}_{2s}\left(\hat{P},\hat{Q}\right)\; & & \;\\ & ={\left(P,Q\right)}^{t}{ℑ}_{2s}\left(P,Q\right)+\; & & \;\\ & \;2{\left(P,Q\right)}^{t}{ℑ}_{2s}\left(\hat{P}-P,\hat{Q}-Q\right)+\; & & \;\\ & \;{\left(\hat{P}-P,\hat{Q}-Q\right)}^{t}{ℑ}_{2s}\left(\hat{P}-P,\hat{Q}-Q\right)\; & & \;\\ & =d\left(P,Q\right)+\; & & \;\\ & \;2{\left(P,Q\right)}^{t}{ℑ}_{2s}\left(\hat{P}-P,\hat{Q}-Q\right)+\; & & \;\\ & \;d\left(\hat{P}-P,\hat{Q}-Q\right)\; & & \;\\ & \; & \end{array}$$

where ${ℑ}_{2s}$ is the 2*s *× 2*s *matrix defined from the identity matrices of dimension *s*, *I_s_*:

(13) $${ℑ}_{2s}=\left(\begin{array}{c} I_{s}\;-I_{s}\\ -I_{s}\;I_{s} \end{array}\right),$$

we have:

(14) $$\begin{array}{c} {\left(\frac{nm}{n+m}\right)}^{1∕2}\left[d\left(\hat{P},\hat{Q}\right)-d\left(P,Q\right)\right]=\\ \;\;2{\left(P,Q\right)}^{t}{ℑ}_{2s}D_{n,m}+\\ \;\;{\left(\frac{nm}{n+m}\right)}^{1∕2}d\left(\hat{P}-P,\hat{Q}-Q\right) \end{array}$$

where *D*_*n*, *m *_has been defined in (10).

Consider first the case *P *≠ *Q*. The second summand on the right-hand side of the above expression tends to zero,

(15) $${\left(\frac{nm}{n+m}\right)}^{1∕2}d\left(\hat{P}-P,\hat{Q}-Q\right)\overset{P}{\underset{n,m\to \infty }{\to }}0$$

while, as a direct consequence of (11), the first summand in (14): is asymptotically normal:

(16) $$2{\left(P,Q\right)}^{t}{ℑ}_{2s}D_{n,m}\overset{d}{\underset{n,m\to \infty }{\to }}U\sim N\left(0,\sigma^{2}\right),$$

with

(17) $$\sigma^{2}=4{\left(P-Q,Q-P\right)}^{t}\Sigma_{PQ}\left(P-Q,Q-P\right).$$

Consider now that *P *= *Q*. Then we have

(18) $$d\left(\hat{P},\hat{Q}\right)=d\left(\hat{P}-P,\hat{Q}-Q\right).$$

From general results on the asymptotic distribution of quadratic forms with a normal basis [24], it can be deduced that

(19) $$V_{n,m}=\left(\frac{nm}{n+m}\right)d\left(\hat{P},\hat{Q}\right)=D_{n,m}^{t}{ℑ}_{2s}D_{n,m}$$

is approximately distributed as a mixture of independent chi-square random variables:

(20) $$V_{n,m}\overset{d}{\underset{n,m\to \infty }{\to }}V=\sum_{i=1}^{s}\beta_{i}\chi_{1,i}^{2},$$

where the *β_i _*correspond to the eigenvalues of the matrix ${ℑ}_{2s}\Sigma_{PQ}$ and the $\chi_{1,i}^{2}$ are independent chi-square random variables with one degree of freedom.

From (16) it follows that under the null hypothesis in (5) (i.e., when *P *= *Q*) $U_{n,m}\overset{P}{\underset{}{\to }}0$. Thus, the decision criterion for (5) may be based on (20).

## Results

In this section we describe two illustrative case-studies and some simulation results on the performance of the statistical methods introduced above.

### Case-studies

We selected two datasets to illustrate our method. The first one is inspired by the work presented in [25], using data kindly provided by those authors. They studied the relationships between phenotypic attributes of a disease and the features of the associated genes, including their ascribed annotated functional classes and expression patterns. The sample gene lists were obtained from the ENSEMBL and OMIM databases and manually curated by the authors. They compared the functional pattern of different groups of genes: (1) genes associated with dominant diseases *vs *genes associated with recessive diseases, (2) genes associated with diseases *vs *all the genes in the human genome. The authors performed their global comparisons using chi-square tests, although they fairly point out that GO classes do not define a true partition of the gene lists or, in other words, that a gene may be annotated in more than one class. Although their conclusions and ours will be similar, we believe our method provides a more appropriate framework for such comparisons. Here we illustrate our method by comparing dominant disease-inducing genes and recessive disease-inducing genes.

**Figure 4:**
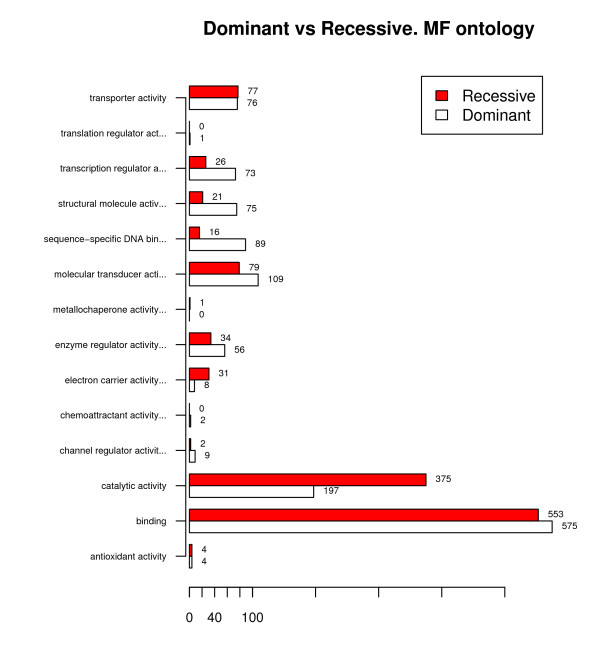
**Dominant vs recessive genes**. Comparison of functional profiles at the second level of the MF ontology based on the lists associated with dominant and recessive diseases.

Table 1 shows the results of applying the global difference test to a list of 985 dominant and 818 recessive genes from the NCBI Entrez database^2 ^projected at the second level of the GO. Figure 4 shows plots of the profiles corresponding to the second level of the molecular function (MF) ontology for dominant and recessive genes. The results of the analysis, which are consistent with those obtained by [25], show that the two sets of genes can be considered functionally distinct with respect to the molecular function (MF) and biological process (BP) ontologies, that is to say, the related dominant and recessive diseases can be associated with different concept categories in both ontologies. With respect to the cellular component (CC) ontology, there are also statistically significant differences although they may be less biologically significant because the profiles are very similar (0.0248 distance).

**Table 1:** Dominant *vs *recessive diseases.

	MF	BP	CC
squared Euclidean distance	0.1029440	0.4138672	0.02482656
p-value	< 2.2 × 10^-16^	< 2.2 × 10^-16^	1 × 10^-4^
95% CI lower limit	0.07004932	0.2715809	0.00894685
95% CI upper limit	0.13583861	0.5561534	0.04070628

Global analysis at level 2.

Results of performing a difference test between functional profiles produced from lists of dominant and recessive genes, at the second level of each ontology. The null hypothesis of equality of profiles can be rejected for all ontologies at a 5% significance level

According to step 1 in our general algorithm, analysis may be continued in order to identify the GO classes associated with the observed global differences. Tables 2 and 3 show the significant GO classes at level 2 for the MF and BP ontologies, respectively. The only significant class for the CC ontology is GO:0032991 with a 4.258815 × 10^-6 ^p-value. The p-values are based on class-by-class analyses by means of Fisher's test, followed by correction for testing multiplicity using the Holm method, [26].

**Table 2:** Dominant *vs *recessive diseases.

Description	GOID	p-value
Binding	GO:0005488	1.591855 × 10^-2^

catalytic activity	GO:0003824	2.847567 × 10^-20^

electron carrier activity	GO:0009055	2.535709 × 10^-3^

sequence-specific DNA binding transcription factor activity	GO:0003700	5.082308 × 10^-14^

structural molecule activity	GO:0005198	2.727443 × 10^-8^

transcription regulator activity	GO:0030528	3.685094 × 10^-6^

Analysis at level 2.

Significant MF level 2 GO classes after a class-by-class analysis based on Fisher's test and correction for multiple testing

**Table 3:** Dominant *vs *recessive diseases.

Description	GOID	p-value
biological regulation	GO:0065007	1.011094 × 10^-13^

cell proliferation	GO:0008283	1.148909 × 10^-10^

death	GO:0016265	4.620938 × 10^-9^

developmental process	GO:0032502	9.242509 × 10^-9^

growth	GO:0040007	3.916801 × 10^-4^

immune system process	GO:0002376	1.032981 × 10^-3^

locomotion	GO:0040011	3.610015 × 10^-4^

metabolic process	GO:0008152	1.654187 × 10^-4^

multi-organism process	GO:0051704	3.214156 × 10^-2^

multicellular organismal process	GO:0032501	2.839762 × 10^-7^

negative regulation of biological process	GO:0048519	1.206870 × 10^-16^

pigmentation	GO:0043473	3.834365 × 10^-3^

positive regulation of biological process	GO:0048518	3.273178 × 10^-13^

regulation of biological process	GO:0050789	2.141995 × 10^-21^

signaling	GO:0023052	9.023421 × 10^-14^

signaling process	GO:0023046	1.113202 × 10^-10^

Analysis at level 2.

Significant BP level 2 GO classes after a class-by-class analysis based on Fisher's test and correction for multiple testing

These differences are also observed in deeper levels of the GO, that is, for more specific categories of molecular functions or biological processes. For illustrative purposes we briefly discuss some results at level 10. For the MF ontology, the global p-value is 0.0001307 but no significant classes are detected when class-by-class analyses are performed in the same conditions as before. However, according to step 3, the ontology class GO:0008094 (DNA-dependent ATPase activity) may be significant. For the BP ontology, a significant global result is also obtained, with a p-value of 8.639 × 10^-8^. The subsequent class-by-class analyses indicate the GO classes in Table 4 as significant.

**Table 4:** Dominant *vs *recessive diseases.

Description	GOID	p-value
negative regulation of transcription from RNA polymerase II promoter	GO:0000122	1.271933 × 10^-2^

negative regulation of transcription, DNA-dependent	GO:0045892	4.613832 × 10^-3^

positive regulation of transcription from RNA polymerase II promoter	GO:0045944	3.114127 × 10^-7^

positive regulation of transcription, DNA-dependent	GO:0045893	5.356749 × 10^-7^

regulation of calcium ion transport	GO:0051924	4.333597 × 10^-3^

regulation of transcription from RNA polymerase II promoter	GO:0006357	2.291754 × 10^-9^

regulation of transcription, DNA-dependent	GO:0006355	9.753411 × 10^-14^

transcription from RNA polymerase II promoter	GO:0006366	8.714318 × 10^-11^

Analysis at level 10.

Significant BP level 10 GO classes after a class-by-class analysis based on Fisher's test and correction for multiple testing

In the second example we compare two microarray experiments described in [27] and [28] to study prostate tumors on the basis of gene expression data. Although the studies were performed independently, the type of tumor they analyzed, the microarray platforms (both studies were based on Affymetrix technology U95AV2) and the sample sizes were all similar; see Table 1 in [29]. Even a substantial proportion of the genes were common to both lists, which makes global comparison methods such as the chi-square test for homogeneity highly inadequate for determining the extent to which these genes represent different functional GO profiles or not. Obviously, the answer to the preceding question may depend on the level of specificity of the GO classes under consideration. The results for very general classes, at level 2 in the GO, are summarized in Table 5. There is only evidence of statistically significant differences (though possibly with little biological relevance, given the very small distances) for the CC ontology, with a 0.004 p-value. When class-by-class Fisher's tests are performed for the CC ontology (this testing step should be avoided for the globally non-significant ontologies, MF and BP) two classes (Table 6) emerge as significant. Significance for the CC ontology is maintained for more specific GO classes. When the analysis is performed at level 10, the global comparison test only produces significant results for the CC ontology, with a p-value of 0.01511. Class-by-class analyses (Table 7) reveal differences in some classes, all related to the ribosome.

**Table 5:** Comparison of two prostate cancer studies.

	MF	BP	CC
squared Euclidean distance	0.001028538	0.004627587	0.003136238

p-value	0.1108498	0.07159675	0.004018912

95% CI lower limit	-5.921965 × 10^-5^	-0.0001544709	0.0004614338

95% CI upper limit	2.116296 × 10^-3^	0.0094096442	0.0058110419

Global analysis at level 2. Results of performing a difference test between functional profiles produced from two studies of prostate cancer ([27] and [28]), at the second level of each ontology. The null hypothesis of equality of profiles can be rejected for the CC ontology at 5% significance

**Table 6:** Comparison of two prostate cancer studies at level 2.

Description	GOID	p-value
organelle	GO:0043226	0.03825311

macromolecular complex	GO:0032991	0.04459121

Significant CC level 2 GO classes after a class-by-class analysis based on Fisher's test and correction for multiple testing

**Table 7:** Comparison of two prostate cancer studies at level 10.

Description	GOID	p-value
cytosolic large ribosomal subunit	GO:0022625	0.0002794424

cytosolic small ribosomal subunit	GO:0022627	0.0028788683

Large ribosomal subunit	GO:0015934	0.0027483186

Small ribosomal subunit	GO:0015935	0.0027483186

Significant CC level 10 GO classes after a class-by-class analysis based on Fisher's test and correction for multiple testing

### Simulations

Simulations were performed in order to assess the validity of the above tests. Their true probability of rejecting the null hypothesis was estimated in different circumstances. Each simulation consisted of the generation of series of 10,000 sample profiles from hypothetical populations whose configurations were suggested (number of GO classes, sample sizes, etc.) by the observed profiles in some selected datasets and studies. The simulated profiles were always generated, in a first step, as "expanded profiles" according to a multinomial distribution, and subsequently converted to "contracted" profiles in order to compute the test statistics. The simulation programs were written in R [30] and executed in 64-bit R 2.12.1 under 64-bit Windows 7 Enterprise edition. An exhaustive simulation study is in process and is the subject of a forthcoming paper. Some early results of this study are available at the above address; they are fully concordant with the preliminary study described here.

Table 8 shows the main results for two simulation scenarios based on [25] and [27,28] and simulating level 10 in the GO. The results in the "true *H*_0_" column correspond to "sample" profiles that were generated from equal "population" profiles, based on pooled data. The results in the "false *H*_0_" column correspond to population profiles directly taken as the observed profiles in the preceding examples (and that to a greater or lesser extent are truly different). For each simulation scenario, the following estimated quantities are displayed:

**Table 8:** Simulation results.

Onto.	*s*	*n*	*m*	A and B gene lists	Testing procedure	*Pr*{*rejectH*_0_} (true *H*_0_)	*Pr*{*rejectH*_0_} (false *H*_0_)
Reference: [25]							

MF	88	69	52	**A **∩ **B **= ∅	Class-by-class	0.0012	0.3903
					
					Chi-square	0.0334	1
					
					New global	0.0469	1
					
					Additional signif. classes	0.04585	0.697

BP	1602	372	328	**A **∩ **B **= ∅	Class-by-class	0.002	1
					
					Chi-square	0.162	1
					
					New global	0.042	1
					
					Additional signif. classes	0.042	0

CC	298	305	336	**A **∩ **B **= ∅	Class-by-class	0.0042	1
					
					Chi-square	0.0775	1
					
					New global	0.0389	1
					
					Additional signif. classes	0.0374	0

References: [27] and [28]							

MF	88	110	99	**A **∩ **B **≠ ∅	Class-by-class	0.0028	0.0729
					
		*k *= 46			Chi-square	0.0341	0.998
					
					New global	0.0428	0.7281
					
					Additional signif. classes	0.0409	0.659

BP	1722	858	651	**A **∩ **B **≠ ∅	Class-by-class	0.003	0.351
					
		*k *= 318			Chi-square	0.152	1
					
					New global	0.056	0.997
					
					Additional signif. classes	0.055	0.646

CC	394	897	679	**A **∩ **B **≠ ∅	Class-by-class	0.0076	0.9982
					
		*k *= 354			Chi-square	0.0883	1
					
					New global	0.0625	0.9999
					
					Additional signif. classes	0.0599	0.0018

Probability of rejecting the null hypothesis of equality of profiles at a nominal 5% significance level in different scenarios associated with real case studies at level 10 in the GO. In the column "testing procedure", "Class-by-class" stands for declaring global differences (i.e. rejecting the null hypothesis of profile equality) if at least one significant class is detected in a class-by-class analysis with correction for testing multiplicity; "Chi-square" stands for the classical chi-square test of homogeneity; "New global" stands for the global test presented in this paper and, finally, "Additional signif. classes" stands for step 3 in the algorithm proposed in the methods section, i.e. proportion of simulation replicates where additional significant classes were detected when a class-by-class analysis was unable of any detection.

• Probability of detecting at least one significant class when a class-by-class analysis with Holm's correction for multiplicity is performed,

• Probability of rejecting the null hypothesis for the standard chi-square test of homogeneity

• Probability of rejecting the null hypothesis for the global test based on (6) and (7), and

• Additional detections of significant classes, according to step 3 of the proposed algorithm, i.e. when no significant classes are detected in a class-by-class analysis but the global test gave a significant result.

All the tests are simulated under a nominal significance level of 0.05.

The classical global chi-square test is clearly incorrect, as may be expected from the arguments in the background section. Its true significance level is very erratic, with very low but also very high values that may largely exceed the nominal level, with an observed maximum of 0.152 for the simulation based on the [25] scenario and the BP ontology.

Also as expected, the new method is at least as powerful (and in general more powerful) than a standard class-by-class analysis. The proportion of true positives that are detected by the class-by-class approach and not by the global test is very low. In the simulated scenarios it ranges from 0 to a maximum of 0.0038 in the simulation scenario inspired by [27,28] and the MF ontology. So, the possible loss in power associated to step 1 in our method is largely compensated by the greater power of the global test.

## Discussion

In this work we present a method for performing global comparisons between groups of genes based on their *functional profiles *that is itself based on their projections at fixed GO levels, or their projections on a set of "interesting" GO classes which could even be at different levels in the ontology.

The method has been shown to perform well in the real case situations analyzed as well as in the simulation studies performed, even for very sparse frequency tables.

We noticed that it has become common practice to perform global tests (that is comparing two lists of genes) based on class-by-class analysis (declaring global differences if there is at least one significant class). Our work suggests that this approach is not appropriate because the dependence between the individual tests for each class and the objective of controlling the FDR or FWER error rates may result in a loss of power. Indeed our simulation results show that this approach yields a less powerful test than the method we present. This is not surprising since making global comparisons is not the main objective of these tests.

Another alternative to performing global tests, the classical homogeneity chi-square test, has also proven not to be valid. On the basis of aprioristic validity reasons explained above in the Background section, and specially in view of its lack of adequate type I error control, its use should be avoided as a tool for making global comparisons between profiles.

Although making a global comparison may often be the main objective of a study, especially if interest is focused on the GO classes that make the difference, the global test may work in conjunction with the usual methods to provide some extra insight. This is particularly clear when many GO classes are considered, for example for deep levels of the GO. Table 8 reflects the true significance level and power of the global test and the class-by-class approach in some scenarios inspired by real examples, at level 10 in the GO.

Even for these cases where thousands of possible GO classes are considered, the global test still has a test size that is near the nominal significance level while at the same time it is more powerful than (or as powerful as) the class-by-class approach.

For those cases where highlighting GO classes causing the difference is of interest, we suggest the following strategy: if class-by-class analysis fails to detect any significant classes but the global test provides a significant result, then highlight as significant the class with the smallest uncorrected p-value. This strategy allows the detection of additional significant classes without inflating the type I error rate.

## Conclusion

In conclusion, the method presented here provides a suitable approach for making global comparisons between lists of genes and should be considered to be complementary to some of the existing ways of comparing lists of genes derived from microarray studies.

In those cases where the user is interested in focusing on a few genes or specific classes, other methods may be more suitable. However, when a global comparison based on the biological meaning of the list of selected genes is required, our method may be the option of choice. It is statistically reliable (Table 8 and http://estbioinfo.stat.ub.es/pubs/goProfiles1_BIF/goProfiles1.htm) and an adequate alternative to the chi-square homogeneity test, which is incorrect to compare GO profiles. Additionally, it may provide some extra insight into GO classes that prove to be interesting but which would not be detected otherwise. This is more apparent at deep GO levels for sparse frequency tables (i.e. profiles), where correcting for a great degree of testing multiplicity imposes a heavy load on the class-by-class approach.

Finally, it is worth mentioning that the applicability of our global comparison method largely surpasses the scope of our conducting examples. As mentioned in the second example (prostate cancer studies), comparison of functional profiles associated with distinct datasets can be used to decide if they can be merged or used combinedly for further studies. Another interesting application appears if one is interested in comparing *gene signatures*, that is groups of genes whose combined expression pattern is uniquely characteristic of a given condition or disease state and which are usually used to characterize or to predict this condition. One problem with signatures is that in many cases there are many signatures for similar situations. A comparison of their associated functional profiles may be used to help deciding if two given signatures are functionally equivalent. Also other useful applications may arise -as kindly reported by a referee - when one is interested in comparing the effect of applying different filtering methods. If two lists of genes obtained by applying different filters, or different cutoffs, do not differ in their functional profiles they might be considered functionally equivalent. Last, although outside the scope of this journal, the method may also have potential applications, like to compare lists of words (e.g. from two literary styles) in terms of their annotation profiles in semantic databases.

## Abbreviations

The following abbreviations have been used along the paper: BP: Biological Process; CC: Cellular Component; EA: Enrichment Analysis; FDR: False Discovery Rate; FWER: Family-Wise Error rate; GO: Gene Ontology; GOID: GO Term Identifier; GSEA: Gene Set Enrichment Analysis; MF: Molecular Function; NCBI: National Center for Biotechnology Information; OMIM: Online Mendelian Inheritance in Man.

## Competing interests

The authors declare that they have no competing interests.

## Authors' contributions

All authors designed the research, AS and MS conceived the approach. MS and JO performed the main Mathematical developments. JO and AS made the program development for the goProfiles package. JO designed and performed the simulation. AS chose and developed the examples. All authors have read and approved the final manuscript.

## Endnotes

^1^Fisher's exact test constitutes nearly a standard in this context and does not require new software development; clearly, a 2 × 2 version of our own test will be a more canonical possibility, but make the method less comparable to the mainstream approach without avoiding the need for a multiple testing correction.,

^2^Given the dynamic nature of the content of biological databases, these lists may have experienced some changes. In order to have a "frozen" version they have not been modified since they were included in the goProfiles package (first version Bioconductor 2.3) so that, in spite of possibly being out of date, they allow the examples in the package to be reproduced.
